# Preliminary evidence of key factors in successful flipping: predicting positive student experiences in flipped classrooms

**DOI:** 10.1007/s10734-022-00848-2

**Published:** 2022-04-08

**Authors:** Erkko Sointu, Mareena Hyypiä, Matthew C. Lambert, Laura Hirsto, Markku Saarelainen, Teemu Valtonen

**Affiliations:** 1 Special Education, School of Educational Sciences and Psychology, University of Eastern Finland, Joensuu, Finland; 2 Department of Special Education and Communication Disorders, University of Nebraska-Lincoln, Lincoln, Nebraska USA; 3 School of Applied Educational Sciences and Teacher Education, University of Eastern Finland, Joensuu, Finland; 4 Department of Applied Physics, University of Eastern Finland, Kuopio, Finland; 5 School of Applied Educational Sciences and Teacher Education, University of Eastern Finland, Joensuu, Finland

**Keywords:** Flipped classroom, Higher education, Learning experience, Quantitative research methods

## Abstract

Flipped classrooms have become widely adopted in educational settings (e.g., in higher education) worldwide. However, there is a need for more precise understanding of the ingredients for student satisfaction in a flipped setting. The aim of this paper was to investigate university students’ experiences of the factors that create a successful flipped course. Ten measures were used to investigate the hypothesized factors affecting satisfaction, which were chosen based on the results from previous flipped classroom studies and higher educational research. These measures were grouped into three dimensions: (1) pedagogical (five measures), (2) social (three measures), and (3) technological (two measures). Exploratory factor analysis was run to analyze the adequacy of the instruments. Results revealed that the factor structure was as expected and that the instruments measuring all ten factors of teaching and learning in a flipped classroom were adequate. Furthermore, confirmatory factor analysis was used to formally operationalize the hypothesized latent constructs, and to build a structural equation model for predicting the student satisfaction of a flipped classroom. In the end, seven factors were found to predict student satisfaction with flipped courses. The highest predictor was guidance from the dimension of pedagogy, and the second-best predictor was experienced teaching for understanding. The results, limitations, and conclusion are discussed in terms of key issues and the development of a flipped classroom pedagogical design for higher education.

## Introduction

Flipped classroom approaches have become widely adopted in educational settings (e.g., in higher education) worldwide (Strelan et al., 2020b). A key feature of the flipped approach is the different sequencing of activities and instruction both before and during class meetings as compared to “traditional” courses (Bergmann & Sams, 2012). Students are expected to prepare themselves for the face-to-face meetings with different pre-materials, such as online video lectures (Talbert, 2017). The face-to-face meetings then focus on facilitating higher-level cognitive activities that usually involve student-centered learning activities, peer instruction, and/or problem solving (Abeysekera & Dawson, 2015), which also emphasize the development of twenty-first century skills (e.g., collaboration and information and communication technology skills). These skills are seen as a necessary component of the contemporary educational process (Valtonen, Sointu, Kukkonen, Häkkinen, et al., 2017a; Voogt & Pareja Roblin, 2012). The flipped approach also includes the use of technology outside of and during class meetings, enabling more time- and place-independent and flexible learning opportunities and various ways for supporting students’ active learning processes (Alexander et al., 2019).

In recent years, the flipped approach has been the focus of several studies. Previous research has outlined students’ perceptions of the approach (Forsey et al., 2013; Mason et al., 2013), learning outcomes of courses conducted with flipped classroom (Ferreri & O’Connor, 2013; Gilboy et al., 2015), and comparisons between flipped and traditional courses (McLaughlin et al., 2014; Strayer, 2012; Tusa et al., 2018). In their meta-analysis, Strelan et al. (2020a) found positive weak–moderate effects on student satisfaction with flipped classroom, indicating that students were more satisfied with the flipped than traditional teaching approaches. Still, the research has provided rather narrow perspectives on the characteristics and success factors of the flipped classroom course. In this study, we attempt to take the next step in examining the educational strategy by using previously accumulated knowledge of higher education and flipped classroom research to develop a more in-depth understanding of the key elements of successful flipped classroom courses from the students’ perspective.

## Background

The results of recent review studies by O’Flaherty et al. (2015) and Akçayır and Akçayır (2018) have shown both positive and negative aspects of the flipped approach. On the positive side, there is strong empirical evidence that using the flipped approach increases students’ academic satisfaction, achievement, and performance (Davies et al., 2013; Strelan et al., 2020a, 2020b). According to Awidi and Paynter (2019), using the flipped approach enhances students’ positive learning experiences, although a more specific analysis of which factors precisely correlate with these experiences is missing (see also Strelan et al., 2020a). Moreover, by emphasizing students’ active learning in a setting where the instructor is available to provide assistance when issues arise, it has proven to be an effective method for supporting deeper learning (Gilboy et al., 2015). From the perspective of flipped classroom, teachers’ pedagogical skills, skills for providing feedback and a safe atmosphere are important for building up student satisfaction, and thus, settings for active learning. Similarly, from the student perspective, possibilities to collaborate, engage in applied activities, and gain support from other students can lead to a more satisfying experience compared to traditional teaching approaches (cf., Parpala et al., 2010; Prince, 2004). However, if students assume a more active role in the learning process, they must, in turn, change their study behaviors as regards, for example, managing their time and taking responsibility during the course (Boevé et al., 2017). Some studies indicate that students may experience frustration regarding the time they have to spend on pre-class activities, especially if the requirements and expectations are unclear during the introduction of the course (Gannod et al., 2008; Mason et al., 2013). Also, because the flipped approach demands self-regulation from students (Lai & Hwang, 2016), students with lower self-regulation skills and higher task avoidance have difficulties in its use (Hyppönen et al., 2019). In addition, Chen et al. (2014) suggests that students who are unable to adapt to the new method fail to familiarize themselves with the pre-class material and, consequently, fall behind in their studies.

As noted by O’Flaherty et al. (2015), there exists no clear model on how to implement the flipped approach into everyday higher education. Nevertheless, some core features for a meaningful flipped classroom course can be identified. Based on recent studies, a good introduction to and guidance on the flipped approach (Mason et al., 2013), a clear structure of learning materials (Hung, 2015), and a consistency between pre-class and in-class activities (Prober & Khan, 2013) contribute to a successful flipped classroom course. Talbert (2017) argues that assessment and feedback given throughout the flipped course are necessary, as students’ efforts are more self-directed compared to a traditionally taught course. Studies by McLaughlin et al. (2014) and Yeung and O’Malley (2014) suggest that giving formative feedback to students helps them to reach higher order cognitive skills. Moreover, Tusa et al. (2018) found that individual feedback provided during the learning process improved students’ learning experience. In addition, more structured guidance and in-time support from teachers (Kim et al., 2014) can lead to better student motivation (van der Velde et al., 2020).

The flipped approach emphasizes the role of the teacher as a dynamic instructor as opposed to a one-way lecturer (Lasry et al., 2014). Student-centered methods pose challenges for teachers, highlighting the importance of teachers’ pedagogical expertise. Hwang et al. (2015) argue that there is a need for teachers to analyze the learning content and objectives to use different teaching strategies in the flipped classroom more meaningfully. This aligns with the description of teachers’ pedagogical content knowledge (PCK; Shulman, 1986), i.e., the knowledge to choose the right pedagogical approaches to meet the specific features of the content being taught to make learning easier and more meaningful for students. Recent studies on the flipped approach have reinforced the significance of teachers’ PCK before and during the course (Bingham, 2011; O’Flaherty et al., 2015). In a flipped classroom, instead of mere content expertise—i.e., broadcasting the contents—teachers need to be able to create learning situations that facilitate collaborative learning activities, to provide support for students with different learning needs. This may cause challenges for teachers who believe that learning is built on transmitting the knowledge, emphasizing a more passive role of students (Kember, 1997; Valtonen, 2011). In this study, the discussed importance of guidance, feedback, teacher’s instructive role, and students’ perceived difficulty during the flipped classroom course will be considered as the pedagogical dimension.

During the face-to-face meetings within the flipped course, collaborative learning activities and peer learning methods are emphasized (Bishop & Verleger, 2013; Talbert, 2017). Discussions in class enhance interactions both between teacher and student as well as between peers. Love et al. (2014) and McNally et al. (2017) found that students had positive views of the collaborative learning activities during face-to-face meetings. Strayer (2012) showed that students were more open to collaborative activities during a flipped course than during a traditional one. Altogether, collaborative activities during the flipped class have indicated better learning outcomes (Kurup & Hersey, 2013) and increased academic performance (Foldnes, 2016). The influence of peers during a flipped course has provided positive support, especially for students with low academic performance (Bergmann & Sams, 2012). However, based on the findings by Kim et al. (2014), merely providing students with collaborative activities during the course did not directly lead to students bonding with each other or to deeper collaboration in the tasks; instead, the teacher’s support and guidance were needed to take advantage of the collaborative activities.

According to Eteläpelto and Lahti (2008), an important element of implementing collaborative learning activities in practice is to build a safe learning atmosphere. Similarly, Baert et al. (2006) indicated openness and safety as important factors in supporting students’ participation and learning. Within a safe and open atmosphere, students are more willing and have greater courage to actively engage in learning activities. In the flipped classroom context, Kim et al. (2014) reported that students experienced the climate in the flipped course as open and safe. Although many previous studies on flipped classrooms highlight the importance of and improvements in student engagement (Ferreri & O’Connor, 2013; McLaughlin et al., 2014), there is scarce evidence of the effects of a safe learning environment during a flipped course. James et al. (2014) see the flipped approach as a well-functioning way to develop safe and encouraging opportunities for learning, while Love et al. (2015) underline the teacher’s role in fostering it. The collaborative and supportive learning, as well as the safe environment, will be considered as the social dimension in the design of this study.

The nature of the flipped approach emphasizing the two entities—prior to class with pre-materials, and face-to-face classes with collaborative and student-centered learning activities—provides several possibilities for fully exploiting information and communication technology (ICT) to support learning. In addition to supporting the actual learning process, the possibilities of ICT provide ways to build a more flexible course, allowing better opportunities for learners with different life situations (Bergmann & Sams, 2012; Lasry et al., 2014). Still, the integration of ICT needs to be considered from the pedagogical perspective, not simply as the use of technology (Abeysekera & Dawson, 2015; Bishop & Verleger, 2013; Kim et al., 2014; Talbert, 2017). So far, there have been some good practices for using ICT to support the flipped courses. Sointu et al. (2019) discovered that students perceived their teaching during a flipped course as having more positive views concerning the use of technology, while McNally et al. (2017) found that students themselves preferred using technology to assist their learning over other aspects in the flipped classroom environment. Moreover, Davies et al. (2013) found that a technology enhanced flipped classroom was more motivating and helped facilitate learning. The relevance of ICT in flipped courses will form the final and third aspect—the technological dimension—in this study.

This theoretical framework provided several important perspectives for the flipped framework. Within this study, we focus on these areas as a framework for a successful flipped course. Currently, there are several flipped classroom studies available, from case studies to larger reviews and meta-analyses. Nevertheless, studies outlining the effects of several factors for a positive flipped course experience are missing. Within this paper, we aim to fill this gap. We see that utilizing theories drawn from research in this area and considering these factors can provide a more comprehensive understanding of the student satisfaction in flipped courses. Thus, our aim is to discover the student perceptions that create a meaningful and successful flipped classroom course.

To fill in gaps in the previous research concerning positive learning experiences in a flipped course in higher education, we outlined the following research questions:

1. What are the factors that predict student satisfaction at the end of the Flipped Classroom course?
2. How and to what extent do the underlying factors explain course satisfaction?

## Methods

### Participants

The convenience sample included 414 (*N*_female_ = 300, 72.5%; *N*_*male*_ = 114, 27.5%) higher education students from the University of Eastern Finland. Slightly more than 56% of participants (*n* = 232) were first-year students, while the remaining students were second-year and older students. The age of participants ranged from 19 to 58 years, with a mean age of 26.19 years (*SD* = 7.58). The data were collected from 24 independent courses taught in the academic years 2016 and 2017. The courses were from different domains in the university, including education, medicine, forestry, and physics. Students responded to the online questionnaire at the end of the course, and the overall response rate between the courses varied from 25% to 75%.

Participation in the study was voluntary for both teachers and students. All participants were informed about elements of their participation in the study, including ethical considerations. Informed consent was obtained from each participant, and the data were protected according to the EU’s General Data Protection Plan (GDPR) and both the national and institutional policies. Non-participation in the study had no effect on the final grading or completion of the course.

### Course design

**Fig. 1:**
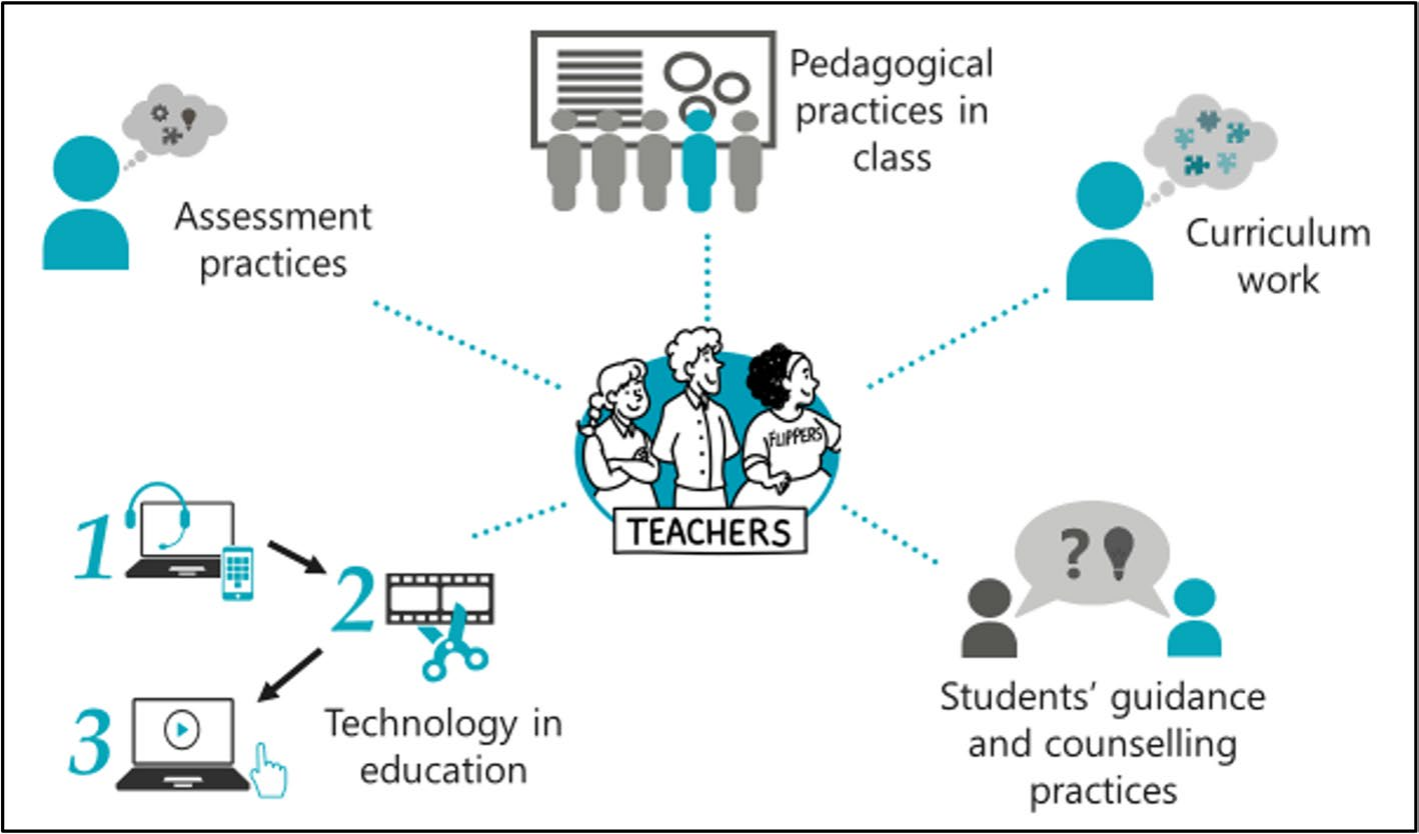
Five key dimensions of the institutional flipped training module

For all participant teachers, the flipped approach was novel, as teachers deployed it for the first time in their teaching practices. Prior to teaching, all teachers took part in a specific institutional flipped approach training module provided by a team of experts responsible for the flipped classroom implementation throughout the university. The team included teachers, researchers, and designers. Training was supported by the institutional strategy and focused on the development of teachers’ skills and curriculum. The key processes of educational development design (e.g., Hirsto, 2021) were used. Teachers received assistance in the form of online video lectures for learning the flipped approach and course planning and implementation, as well as supportive material, individual and peer group support, tutoring, and information on the data collection for practice-based research (Sointu et al., 2021). The support was available for teachers throughout the flipped implementation. The institutional flipped training was organized through five dimensions (Fig. 1), all of which were carefully chosen based on the previous research and experiences obtained from the flipped context.

The foundation of the training, *curriculum work*, aimed at helping teachers to read over the content of the course and to consider how to provide the content as pre-materials, typically in the form of a series of short video clips instead of lectures. This also guided the teachers in the entire process of planning, designing, developing, implementing, and assessing the courses. Building on the curriculum work, the next section was *pedagogical practices in class*, containing information and instruction on the teacher’s role in the flipped approach. It included considerations on, for example, how to organize the face-to-face sessions and activities to support more active learning by students. As technology is widely used in the flipped approach, a strong emphasis was placed on *technology in education* throughout the training. Not only did the teachers develop practical knowledge on the use of various applications and devices, there were also profound discussions on the benefits of technology for both teaching and learning. Furthermore, *students’ guidance and counseling practices* informed teachers’ instruction regarding how to familiarize and support students with the novel study method. Finally, *assessment practices* highlighted the use of formative assessment and its benefits on student learning and achievement. Teachers were provided concrete examples on practices of formative assessment during the training. All five sections incorporate factors of a positive flipped classroom learning experience, as discussed in the theoretical background section of this article. It is important, however, to consider that the aim of this study was to investigate students’ perception of the flipped classroom; consequently, the teacher’s pursued goals are not always perceived similarly by the students. Therefore, this study seeks to identify the underlying factors of a positive flipped classroom learning experience from students’ perspective to further improve the quality of higher education.

### Measures

To align with the results of previous flipped classroom research themes, we benchmarked existing instruments for these research areas and selected the most appropriate ones. While most of the dimensions were measured by these already existing research instruments (i.e., translated into Finnish and psychometrically sound), some had to be developed and pilot tested for the purposes of this study. Students’ experiences of flipped classroom were measured from several different perspectives in three sections. The first section focused on the pedagogical dimension and consisted of the following five instruments: (1) their teachers’ pedagogical content knowledge (3 items, e.g., *“My teachers use versatile teaching methods in order to make the studied matter easily understandable”*; Valtonen, Sointu, Kukkonen, Kontkanen, et al., 2017b); (2) pedagogical perceptions about teaching that is aimed at understanding (4 items, e.g., *“The teachers helped students to observe the thinking process relevant to the discipline and how to reach to conclusions”*); (3) constructive feedback (3 items, e.g., *“The feedback I received from the course assignments helped me to clarify matters I had not fully understood”*) obtained during the course; (4) the level of experienced difficulty of flipped classroom (3 items, e.g., *“It took a lot of time to familiarize oneself with the pre-class material”*) during the course; and (5) guidance for the flipped classroom as a study method (5 items, e.g., *“During the course, students were adequately instructed on the study method in use”*). Understanding and feedback instruments came from the Experiences of Teaching and Learning Questionnaire by Entwistle et al. (2002), and variables for difficulty and guidance instruments were created specifically for this research. The second section measured the social dimension and investigated students’ perceptions of (1) collaborative working (5 items, e.g., *“Studying in groups improves the quality of my learning”*; Wang et al., 2009); (2) support from other students (3 items, e.g., *“The students supported each other and tried to help when it was needed”*; Entwistle et al., 2002); and (3) safe atmosphere for learning (3 items, e.g., *“The course had a safe atmosphere to ask things and to question the contents discussed during the lessons”*) with an instrument created specifically for this study. Finally, in the third section, students’ perspectives on the technological dimension were studied with the following two instruments: (1) students’ readiness to use information and communication technology (ICT) for studying (4 items, e.g., *“I know how ICT is used effectively to support learning”*; Valtonen, Sointu, Kukkonen, Kontkanen, et al., 2017b); and (2) how students experienced the added value of ICT in education (4 items, e.g., *“The use of ICT increases my chances of gaining more skills”*; Chen, 2011). Pedagogical content knowledge, difficulty, guidance, collaboration, readiness to use ICT, added value of ICT, and satisfaction were measured with an original Likert-type scale (1 = Totally disagree, 6 = Totally agree). Understanding, feedback, support, and safe environment were measured with an original Likert-type scale (1 = Never, 5 = Always).

The *Satisfaction with flipped classroom* variable was created for this study. It consisted of the following five items: “*In my opinion, the course functioned well as a whole*”; “*Pre-class materials and contact teaching complemented each other well*”; “*Pre-class materials and contact teaching as a whole provided an effective framework for learning the content matter*”; “*The use of pre-class materials effectively helped to prepare for contact teaching*”; and “*The study methods used in contact teaching helped understand more comprehensively the topics of pre-materials.*” The satisfaction with flipped classroom was used as the outcome variable in the analyses.

### Data analyses

To analyze how different aspects predict the experienced course satisfaction, we used a two-step procedure. *First*, exploratory factor analysis (EFA) was used to preliminarily investigate the factor structure of the constructs because these measures had never been factor analyzed before. While there were a priori hypothesized factor structures to each measure (see the *Measures* section), an initial EFA seemed prudent given the lack of empirical data on the factor structures. EFA was analyzed in four sections corresponding to the four construct dimensions: (a) 18 items for the pedagogical dimension, (b) 11 items for the social dimension, (c) eight items for the technological dimension, and (d) four items for satisfaction with flipped classroom. Principal axis factoring model with a direct oblimin rotation was used for factor extraction. Eigenvalue (>1) and scree plot were used for factor interpretation, and the Kaiser–Meyer–Olkin measure of sampling adequacy (> 0.8) and Bartlett’s test of sphericity (*p* < 0.01) were used to assess the suitability for EFA solutions. Because instruments were measured using the different response categories, variables were standardized as z-scores before EFA.

*Second*, we specified a structural equation model (SEM) to investigate how student experiences of the flipped course predict satisfaction with flipped classroom. The SEM was built in three stages: (a) a confirmatory factor analysis (CFA) model was specified to operationalize the pedagogical, social, and technological constructs based on a priori hypothesized factor structures; (b) the CFA model was evaluated for mis-specification (e.g., Heywood cases, high structure coefficients) and modified accordingly; and (c) the CFA model was extended to include regression parameters to predict satisfaction with flipped classroom.

SPSS v25 was used for the EFA models. Mplus v8.1 (Muthén & Muthén, 1998–2019) was used to fit the CFA and SEM to the item-level data. We used the weighted least squares with mean and variance adjustments estimator for both the CFA and SEM because the item-level data were ordinal Likert-type ratings and demonstrated non-normal distributions, which violates the multivariate normal assumption of maximum likelihood (Flora & Curran, 2004). Mplus provides several indicators of goodness-of-fit: chi-square (χ^2^), comparative fit index (CFI; Bentler, 1990), the root mean square of approximation (RMSEA; Steiger & Lind, 1980), and the standardized root mean square residual (SRMR; Hu & Bentler, 1999). Because chi-square can be overly sensitive to sample size, we relied on CFI, RMSEA (and its 90% confidence interval), and SRMR to evaluate the model fit. Generally, for close model fit, CFI should be above .95, and for an acceptable model fit, between .90 and .95 (Hu & Bentler, 1999). RMSEA and SRMR < .06 are considered excellent, while values between .06 and .08 are acceptable (Brown, 2015; Hu & Bentler, 1999).

When evaluating the regression coefficients of the SEM, we reported and interpreted the fully standardized coefficients (i.e., STDYX coefficients), which represent the change in the outcome variable (in standard deviation units) for a one standard deviation change in the predictor, while holding other predictors in the model constant. These coefficients are interpreted in the same way as standardized “beta” (*β*) coefficients from a linear multiple regression analysis. Because there were ten comparisons of interest in this study, there is potential for an inflated type I error rate. Therefore, we set the per-test significance level to .005, which represents a conservative adjustment that maintains a nominal type I error rate of .05 across the entire set of ten comparisons.

To aid in the interpretation and comparison of regression coefficients, we also computed Pratt measures (Pratt, 1987) for regression coefficients, which indicate the relative importance and contribution of each predictor in the model (Thomas et al., 1998). Specifically, Pratt measures indicate the proportion of explained variance that is attributable to each predictor in the model. Intuitively, Pratt measures should be positive in sign. Negative Pratt measures indicate some problematic characteristic of the predictor—either multicollinearity or a suppressor effect (Thomas et al., 1998). In either case, a negative Pratt measure helps to identify a predictor that does not contribute uniquely to predicting of the outcome variable.

## Results

### Exploratory factor analysis

EFA was undertaken to provide an initial basis for further factor analysis using a confirmatory framework. Based on the EFA, ten factors were identified and named accordingly to describe the different aspects of a student’s experience in the flipped classroom course. Factors, number of items, loadings, and reliability (Cronbach’s coefficient alpha) of the factor are presented in Table 1. Five of the factors (pedagogical content knowledge, understanding, feedback, difficulty, guidance) were about the pedagogical dimension, three (collaboration, support, safe environment) were about the social dimension, and two (readiness to use ICT, added value of ICT) were about the technological dimension. Satisfaction with the flipped classroom was analyzed separately, as it was a predictor in the later analysis.

**Table 1:** Exploratory factor analysis and internal consistency (reliability)

	Rotated factor solution		
	N of items	Loadings	Cronbach’s *α*
Pedagogical dimension ^a^			
Students’ view of their teachers’ pedagogical content knowledge	3	-.84 to -.75	.84
Pedagogical perceptions about teaching that is aimed at understanding	4	.70 − .81	.84
Constructive feedback	3	-.90 − .54	.80
Level of experienced difficulty of flipped classroom	3	.64 − .90	.78
Guidance for the flipped classroom as a study method	5	.59 − .89	.86
Social dimension ^b^			
Collaborative working	5	.45 − .87	.85
Support from other students	3	.70 − .78	.83
Safe atmosphere for learning	3	.53 − .92	.82
Technological dimension ^c^			
Students’ readiness to use ICT for studying	4	-.91 to -.60	.88
Added value of ICT in education	4	.72 − .82	.85
Outcome variable ^d^			
Satisfaction with flipped classroom	5	.50 – .81	.89

^a^Cumul. variance extracted 71.9%; KMO –.860; BTS *x*^2^ = 3559.414; df = 153; *p* ≤ 0.001

^b^Cumul. variance extracted 70.8%; KMO – .846; BTS *x*^2^ = 2204.423; df = 55; *p* ≤ 0.001

^c^Cumul. variance extracted 71.3%; KMO –.876; BTS *x*^2^ = 1759.479; df = 28; *p* ≤ 0.001

^d^Cumul. variance extracted 71.1%; KMO – .872; BTS *x*^2^ = 1171.621; df = 10; *p* ≤ 0.001

All extracted factors in the pedagogical and technological dimensions had eigenvalues greater than 1, and all item loadings exceeded 0.50. The initial EFA for social dimension extracted only two factors, removing items with eigenvalues less than 1. However, examination of the inflection point of the scree plot indicated that three factors should be extracted (Cattell, 1966). In addition, the eigenvalue (.93) was just slightly below the preferred value 1. Upon examining the scree plot and considering both the eigenvalue and the factor solution in relation to the learning environmental theory, the most meaningful solution was to interpret the social dimension as a three-factor solution.

### Structural equation model

**Fig. 2:**
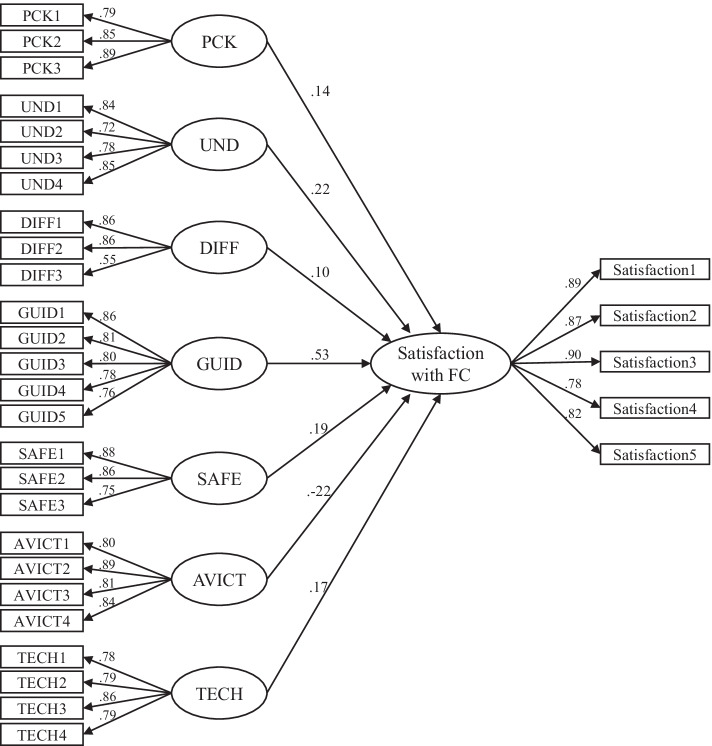
The final SEM *Note,* only factors that significantly predicted satisfaction with flipped classroom are shown. *PCK* Students’ view of their teachers’ pedagogical content knowledge, *UND* Pedagogical perceptions about teaching that is aimed at understanding, *DIFF* Level of experienced difficulty of flipped classroom, *GUID* Guidance for the flipped classroom as a study method, *SAFE* Safe atmosphere for learning, *AVICT* Added value of ICT in education, *TECH* Students’ readiness to use ICT for studying, *FC* flipped classroom

Following the initial EFA, a CFA was fit to the item-level data. The aim of the CFA was to operationalize the constructs in a confirmatory framework. After the initial CFA model for pedagogical, social, and technological dimensions was specified (without satisfaction with flipped classroom), the model was modified in two ways. The initial CFA indicated a negative residual variance for two items (i.e., Heywood cases). A common solution to this issue is to fix the factor loading for items to one. A revised CFA with the factor loadings for the problematic items fixed to one solved the issue and resulted in an acceptable fit to the data: χ^2^_(585)_ = 1597.76, CFI = 0.93, RMSEA .065 [.061, .068], SRMR = 0.05. After this, the measurement model was expanded to include structural parameters to estimate the extent to which pedagogical, social, and technological factors predicted satisfaction with flipped classroom. The final SEM fit the data acceptably well: χ^2^_(765)_ = 1923.28, CFI = .94, RMSEA = .060 [.057, .064], SRMR = 0.05. The final SEM is diagramed in Fig. 2.

Of the ten latent factors operationalized in the CFA model, seven significantly predicted satisfaction with flipped classroom when evaluated at the .005 significance level (see Fig. 2). In total, the model explained 82.3% of the total variance of the satisfaction latent factor. Of the factors that significantly predicted satisfaction with flipped classroom, guidance (*β* = 0.53) was the most important predictor and accounted for nearly 52% of the explained variance. The second most important predictor was pedagogical perceptions about teaching aimed at understanding (*β* = 0.22), which accounted for 19% of the explained variance; this was followed by safe atmosphere for learning (*β* = 19; 16.8% of explained variance), students’ view of their teacher’s pedagogical content knowledge (*β* = 0.14; 11.3% of explained variance), and students’ readiness to use ICT for studying (*β* = 0.17; 8.9% of explained variance). While the level of experienced difficulty in flipped classroom (*β* = 0.10) and the students’ perception of added value of ICT in education (*β* = -0.22) significantly predicted satisfaction, neither factor contributed uniquely to the prediction of satisfaction (i.e., both factors had a negative Pratt measure, which indicates that neither factor predicted unique variance). Because both were significant predictors of satisfaction, these factors may be important suppressor variables for future research to study further. Feedback, collaborative working, and support from other students did not significantly predict satisfaction.

## Discussion

In this study, we reported factors that predicted student satisfaction at the end of the flipped classroom course. The aim was to provide a comprehensive picture of the factors that create a successful flipped course based on students’ experiences from several courses. Initially, ten measures were used to investigate the hypothesized factors affecting satisfaction, all of which were chosen based on the results from previous flipped classroom studies. Exploratory factor analysis was conducted to provide an initial factor analysis of the assessment data. The results revealed that the factor structure was as hypothesized and measured all ten factors of teaching and learning in a flipped classroom. Furthermore, the confirmatory factor analysis was used to formally operationalize the hypothesized latent constructs, and the structural equation model indicated that seven of the ten factors significantly predicted student satisfaction with flipped courses. These findings underscore the importance of student satisfaction with pedagogical approaches (e.g., flipped classroom), which should not be underestimated by teachers nor institutions (Strelan et al., 2020a).

From the perspective of pedagogy, guidance was strongly predictive, accounting for over half of the explained variance in satisfaction with a flipped classroom. This finding suggests a need for clear and understandable instructional materials, along with the importance of thoroughly explaining the flipped classroom learning practices for students—i.e., what it means to study within a flipped course and what is expected from them. Also, teaching that aims for understanding by facilitating students in generating a comprehensive understanding of both the content being taught and the discipline more generally led to positive satisfaction with the flipped approach. This factor accounted for nearly one-fifth of the explained variance. Along with these factors, teachers’ pedagogical content knowledge predicted satisfaction with flipped classrooms. We assume that the pedagogical content knowledge acted as an overall assessment of teachers’ abilities to conduct a flipped course, reflecting the areas of previously mentioned factors of pedagogy. Thus, students saw their teacher as being competent to choose appropriate pedagogical approaches to teach certain content areas. Finally, the difficulty level of the flipped approach had an impact on satisfaction as well; however, the effect was small and did not contribute uniquely to predicting satisfaction.

Flipped classroom as a learning approach demands students to take more control over their own learning process, for example, in planning the time used for pre-class activities and going through the pre-class material well enough to be able to deepen their knowledge in the class, which might be new and challenging for the students. A flipped classroom can promote students’ self-regulation (Lai & Hwang, 2016), but helping students to shift from being passive to active learners requires sufficient guidance. It also seems that self-regulation functions differently with various students (e.g., Hyppönen et al., 2019); thus, the highly emphasized role of guidance and instruction for all students is quite understandable. These results align with previous studies by Mason et al. (2013), whose results showed the importance of good course introduction and guidance. Similarly, the study by Hung (2015) found that the pre-materials should be easy to understand, well-structured, and clearly presented. Altogether, these findings align with observations by Boevé et al. (2017), who highlight the need for assisting students in the change of adapting their study behavior to the novel study method. Hence, these strongly suggest that guidance can predict a positive flipped course experience.

The social dimension was covered using three factors; however, only one—the safe atmosphere—explained students’ positive experiences of the flipped course. This result aligns with previous studies suggesting that a flipped classroom can be seen as a way of creating a safe environment for learning (see James et al., 2014; Kim et al., 2014). The flipped approach is based on collaborative activities, with receiving and providing help and discussing through difficult topics. Consequently, a safe environment encourages students to engage in these activities, to ask questions, and to interact with their peers and teachers more openly, without a fear of being ridiculed (see Eteläpelto & Lahti, 2008), thus resulting in a positive course experience. These results support the outcomes of the study by Love et al. (2015), indicating that the teacher’s role is important in fostering a safe learning environment to make the core learning activities of a flipped classroom possible.

Interestingly, the role of information and communication technology (ICT) had mixed effects on the flipped course experiences: although the students’ readiness to use ICT was significant and positively related to the positive experiences, the added value of ICT in education did not uniquely contribute to the predictive model. Typically, flipped courses contain several ICT applications and software, such as platforms for providing the pre-materials and assignments alongside tools for supporting learning in face-to-face meetings (Talbert, 2017). This makes the results partly plausible: the students who are confident in their ICT skills can be assumed to perform well within the technology-rich flipped environments. However, the negative relation between the added value of ICT and satisfaction appears to be a methodological artifact. The zero-order correlation between the added value of ICT and satisfaction was positive, yet the regression coefficient was negative; this suggests either an issue with multicollinearity or a suppression effect. In either case, we cannot infer a substantive interpretation of the relation in this study; however, the relation between ICT and satisfaction remains an interesting topic for future studies.

From the student’s perspective, ICT skills are needed to cope with various technologies used in a flipped course, both online and face-to-face. Additionally, access to ICT may be an issue. However, access to technologies (e.g., networks, devices, and skills) is typically readily available to Finnish higher education students, and higher education institutions provide additional possibilities for technology access with free internet connections on campus and devices (e.g., students’ working spaces) (e.g., Digivision 2030, 2021). Interestingly, the factors which did not directly predict the positive course experience focused on feedback, collaborative working, and support from other students. We assume that, especially from the perspective of collaboration, it may be that the students were not able to fully take advantage of the collaborative learning situations for their learning needs, or the collaborative activities did not serve the learning goals set for the course. These results indicate that the role of collaboration related to satisfaction may have served a minor role in the course satisfaction compared to the expectations underlined within previous studies of flipped classroom (O’Flaherty et al., 2015).

The results of this study align with previous findings, reinforcing the importance of the teacher’s role within the flipped courses (Bingham, 2011; O’Flaherty et al., 2015). This study highlights the importance of clearly outlining the learning process of a flipped classroom for the students and the need for providing students with clear instruction and guidance. Also, as the flipped approach may be new for students, the more self-directed role of the students may cause challenges and pose demands for more support (e.g., Hyppönen et al., 2019; Sointu et al., 2019). Again, students need to be provided with the feeling that they are learning in a safe atmosphere as well as with a secure feeling for participating, bringing up their ideas, and asking for help whenever needed. This aligns with previous studies from the perspective of face-to-face collaboration (Bishop & Verleger, 2013). These elements pose demands for the teachers’ role as the expert of pedagogy, particularly in their ability to design and maintain an environment and an atmosphere that meet these demands.

These demands, however, can be challenging for the teachers to meet, as the teaching staff in the higher education context may not hold much pedagogical training or experience. O’Flaherty et al. (2015) argue that educators’ scarce pedagogical understanding prevents the flipped approach from being effectively translated into practice and therefore limits the possibilities of a flipped classroom for curriculum renewal. In this study, teachers were prepared in the approach via institutional flipped training that addresses the approach as a whole and thus allows an effective organization-level curriculum shift. As Wanner and Palmer (2015) found the absence of institutional support for flipped classroom to be one of the main concerns of higher education teachers, providing institutional support is therefore crucial for a successful deployment of flipped classroom. The findings of this study further highlight which factors contribute to student satisfaction. We suggest that our findings provide student-derived guidelines for flipped teacher training to improve the curriculum shift from the theoretical flipped classroom model to the actual well-functioning and effective flipped class, and consequently, can serve as guidelines for national or local institutions in policy and practice development.

## Limitations

While our study sample was relatively large as compared to other research related to flipped classrooms, the participants were sampled from only one institution, and therefore, the generalizability of the findings may be limited. Future research should consider selecting samples from multiple institutions that better represent the target population. This study focused on students’ perceptions of their experiences of a positive flipped course; however, we were not able to collect comparable data for students who were enrolled in courses taught using a more traditional approach. A future study could therefore assess which factors affect learning experiences in general, regardless of the instructional approach, and whether those relations differ between instructional approaches. A future study could also explore the extent to which the relations between students’ perceptions and satisfaction are moderated by discipline or area of study. It is important to gain a more nuanced understanding of how characteristics of flipped classrooms may vary across disciplines and how those characteristics may relate to satisfaction differentially across disciplines.

As with any study using predictive modeling approaches, it is difficult to include an exhaustive set of potential predictors of the targeted outcome. Therefore, it is possible that our model omitted important predictors of satisfaction with the flipped classroom, such as univocal measures of active learning, cognitive load, or self-determination. The omission of key variables could bias the results of the predictive model by either over- or underestimating the magnitude of the relations between student experiences in flipped classrooms and student satisfaction with flipped classrooms. While the findings of this study are substantive and align with prior research on flipped classrooms, the results should be viewed as an initial step toward a better understanding of how student experiences in flipped classrooms relate to student satisfaction, and future research on this topic should consider many of the factors included in this study, but also additional factors not included in this study (e.g., direct measure of active learning) to develop a more complete understanding of student satisfaction with flipped classroom.

Since our study underlined the importance of guidance in the flipped approach, we believe that it opens an interesting avenue for further research. These studies could more closely examine the characteristics of guidance and instruction during a flipped course, and thus shed light on timing, quantity, approach, and other factors of guidance influencing the learning experience. Moreover, an in-depth investigation of the technology should be considered. It is necessary to investigate and define design specifications that integrate technology meaningfully into flipped classrooms. In particular, COVID-19 changes to the pedagogy and technology should be investigated. An additional limitation is related to the research method used in this study. Specifically, this study did not investigate possible teacher effects or longitudinal perspectives, nor did this study account for any of the levels of nesting that were inherently present in the data. In the future, researchers should consider using multilevel modeling approaches (for either nested or longitudinal data) for both sound methodological and substantive reasons. Despite the limitations, a clear conclusion based on the research can be made.

## Conclusion

Altogether, the findings of this study provided new understanding concerning the nature of a successful course conducted using the flipped approach in higher education. The results combine findings from previous studies and show their relation to the positive course experiences on a large scale. Our findings show that the flipped approach needs to be considered as a method that demands effort in outlining the aims of the flipped approach clearly for the students, using clear and comprehensive instructions throughout the course, and designing and delivering contents that allow students to focus on the key points of the course within a safe environment. Consequently, these results can be assumed to reflect students’ twenty-first century skills, as self-directed learning in collaboration with peers using technology are key elements of these skills (Voogt & Pareja Roblin, 2012). These themes also reflected from the results the success factors of the flipped courses. We suggest these results can be seen as a way to develop higher education flipped courses and with that, contribute to students’ twenty-first century skills.

## Data Availability

Not available as under investigation by the research group.
